# Genetic analysis of 106 sporadic cases with hearing loss in the UAE population

**DOI:** 10.1186/s40246-024-00630-8

**Published:** 2024-06-07

**Authors:** Abdelaziz Tlili, Mona Mahfood, Abdullah Al Mutery, Jihen Chouchen

**Affiliations:** 1 Department of Applied Biology, College of Sciences, University of Sharjah, Building W8 Room 107, P.O. Box: 27272, Sharjah, United Arab Emirates; 2 Human Genetics and Stem Cell Laboratory, Research Institute of Sciences and Engineering, University of Sharjah, Sharjah, United Arab Emirates

**Keywords:** *GJB2 screening*, Whole exome sequencing, Hearing loss, DNA variations, Diagnostic rate

## Abstract

**Background:**

Hereditary hearing loss is a rare hereditary condition that has a significant presence in consanguineous populations. Despite its prevalence, hearing loss is marked by substantial genetic diversity, which poses challenges for diagnosis and screening, particularly in cases with no clear family history or when the impact of the genetic variant requires functional analysis, such as in the case of missense mutations and UTR variants. The advent of next-generation sequencing (NGS) has transformed the identification of genes and variants linked to various conditions, including hearing loss. However, there remains a high proportion of undiagnosed patients, attributable to various factors, including limitations in sequencing coverage and gaps in our knowledge of the entire genome, among other factors. In this study, our objective was to comprehensively identify the spectrum of genes and variants associated with hearing loss in a cohort of 106 affected individuals from the UAE.

**Results:**

In this study, we investigated 106 sporadic cases of hearing impairment and performed genetic analyses to identify causative mutations. Screening of the *GJB2* gene in these cases revealed its involvement in 24 affected individuals, with specific mutations identified. For individuals without *GJB2* mutations, whole exome sequencing (WES) was conducted. WES revealed 33 genetic variants, including 6 homozygous and 27 heterozygous DNA changes, two of which were previously implicated in hearing loss, while 25 variants were novel. We also observed multiple potential pathogenic heterozygous variants across different genes in some cases. Notably, a significant proportion of cases remained without potential pathogenic variants.

**Conclusions:**

Our findings confirm the complex genetic landscape of hearing loss and the limitations of WES in achieving a 100% diagnostic rate, especially in conditions characterized by genetic heterogeneity. These results contribute to our understanding of the genetic basis of hearing loss and emphasize the need for further research and comprehensive genetic analyses to elucidate the underlying causes of this condition.

## Introduction

Hearing loss (HL) is an etiologically heterogeneous sensory deficit with genetic predisposition being a key factor in most congenital cases [1]. Although next-generation sequencing (NGS) panels and whole exome sequencing (WES) have been instrumental screening approaches during the past decade, the complexity of hearing loss genetics continues to be a diagnostic obstacle. Excluding genes involved in syndromic forms of HL, over 120 genes have been linked to non-syndromic HL so far (https://hereditaryhearingloss.org/). The reported mutational spectrum associated with these genes mainly includes point mutations, insertions and deletions (indels) as well as copy number variations (CNVs) [2, 3]. This genetic diversity often creates phenotypic variability among patients, which further complicates the diagnostic process. This is particularly evident when different mutations in the same gene lead to varying hearing loss severities, or when the same mutation results in intra-familial variability [4–6]. Phenotypic variability is also seen in some genes linked to both syndromic and non-syndromic HL such as *MYO7A*, and *CDH23* [7, 8]. Additionally, while most HL genes have distinct inheritance patterns, several genes have been linked to more than one mode of inheritance. These issues collectively make the establishment of genotype-phenotype correlations often challenging [9, 10].

The use of NGS approaches for HL diagnosis is also hindered by low coverage in regions with high GC content, homology, and DNA complexity as well as limitations in our knowledge of the genome [11]. This is especially important for the detection of CNVs in genes such as *STRC* and *OTOA* which have very homologous pseudogenes. These regions of high homology greatly impact variant calling and lead to recurrent deletions and duplications as a result of non-allelic homologous recombination [2, 12, 13]. Therefore, combining NGS with additional CNV detection strategies such as multiplex ligation-dependent probe amplification (MLPA), droplet digital PCR, chromosomal microarray, and allele-specific PCR is often recommended to improve diagnostic accuracy [14].

Ensuring that a mutation completely segregates with the HL phenotype is another important aspect of making a conclusive genetic diagnosis. However, in many cases the unavailability of family history, lack of samples from important family members as well as segregation of the suspected variant with only some of the affected family members represent additional diagnostic hurdles [15]. Moreover, the emergence of cases displaying digenic inheritance where more than one pathogenic mutation appears to segregate with the HL phenotype has also been a point of discussion in recent years. The presence of digenic cases may reflect the multiple roles played by genes critical to the hearing process, as well as their ability to interact or perform co-dependent functions [16–18]. Nonetheless, limited knowledge of the auditory mechanism’s molecular control highlights the necessity for further functional studies. These studies can verify digenic claims and clarify the impact of mutations, specifically in the case of ambiguous missense and UTR variants.

In this study, 106 sporadic HL cases from the UAE population were screened for mutations using a combination of Sanger sequencing and WES. Though our screening approach reinforced the important contribution of *GJB2* mutations among hereditary HL patients in the UAE, it also revealed relevant variants in over 20 other HL genes. Despite these findings, many cases remained undiagnosed, underscoring the intricacy of HL genetics and the shortcomings of WES when used as the sole diagnostic approach.

## Materials and methods

### Sample collection and *GJB2* gene screening

One hundred and six sporadic cases with congenital hearing loss, primarily residing in Sharjah, Dubai, and Al Ain, were recruited from three different organizations for the Deaf and Hard of Hearing in the UAE. The recruitment period spanned from October 11, 2021, to February 23, 2023. Informed written consent was obtained from all participants and their genomic DNA was extracted from their saliva using the Oragene-DNA (OG-500) Kit (DNA Genotek, Canada). This study was approved by the Sharjah Research Ethics Committee at the University of Sharjah, Sharjah, United Arab Emirates. All affected individuals were screened for *GJB2* variants using Sanger sequencing as previously described [19].

### Determining cis/trans configuration of *GJB2* variants

To check the cis/trans configuration of c.235delC and c.299_300delAT *GJB2* variants, we cloned the PCR product of the sample showing these two variants in the heterozygous state into the pGEM-T Easy vector (Promega, USA) according to the manufacturer instructions and recombinant clones were then analyzed by Sanger sequencing.

### Whole exome sequencing

Affected individuals with no *GJB2* mutations were analyzed by WES using the Illumina HiSeq 2500 system as previously described [20]. In summary, exome was captured using the SureSelect All Exon V5 kit. The resulting reads passing quality control were mapped to the human reference genome, and variant calling was performed using Genome analysis tool kit (GATK) v2.7.2. Variants were annotated and filtered based on read depth and frequency in various databases. Finally, the functional impact of candidate variants was predicted using several bioinformatics tools including Variant Effect Predictor (VEP), Mutation Taster, VarSome, PROVEAN, PolyPhen-2, SIFT, and Human Splicing Finder.

### Sanger sequencing.

To screen the *GJB2* gene and validate variants detected by WES and recombinant plasmids, Sanger sequencing was performed. In brief, after PCR amplification, amplicons were treated with ExoSAP-IT PCR Product Cleanup Reagent (78200.200.UL, Applied Biosystems, Thermo Fisher Scientific, USA) and subsequently used in the sequencing reactions conducted using the BigDye Terminator v3.1 Cycle Sequencing Kit (4337455, Applied Biosystems, Thermo Fisher Scientific, USA). After purification, the sequencing reactions were analyzed using the Genetic Analyzer 3500 (Applied Biosystems, Thermo Fisher Scientific, USA).

## Results

**Fig. 1:**
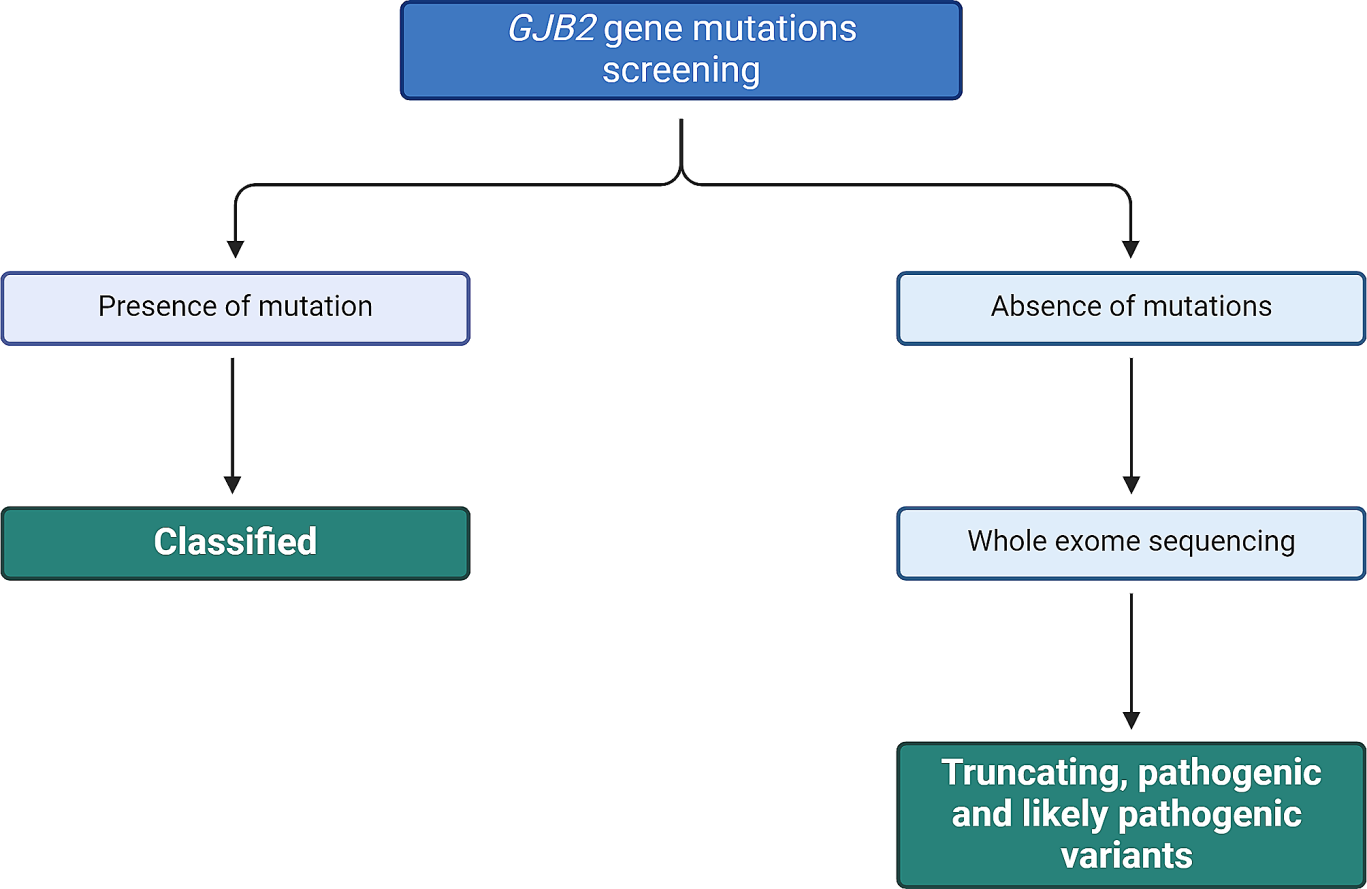
Screening cascade used in this study. This figure illustrates the study’s screening process: All 106 hearing loss cases were initially screened for *GJB2* gene mutations. Cases with detected mutations were classified accordingly. In the absence of mutations, further whole exome sequencing was conducted, and the resulting DNA variants were selectively analyzed to identify those that are rare, truncating, or classified as pathogenic or likely pathogenic within HL genes

Screening of the *GJB2* gene in 106 sporadic cases revealed the involvement of this gene in 24 affected individuals (Fig. 1). In fact, 20 cases were homozygous for the c.35delG (p.Gly12Valfs*2) mutation, 3 homozygous for the c.506G > A (p.Cys169Tyr) and one patient was heterozygous for the c.235delC (p.Leu79Cysfs*3) and c.299_300delAT (p.His100Argfs*14) pathogenic variants (Table 1). Given the absence of his parents’ DNA samples, we cloned the PCR product into the pGEM-T Easy vector and found after sequencing recombinant clones that c.235delC and c.299_300delAT are in trans. These results confirmed the implication of these two truncating mutations in the hearing loss phenotype observed in this affected individual.

**Table 1:** Screening Results of *GJB2* Gene Mutations in 106 HL Sporadic Cases

Mutation	Zygosity	Number of cases
c.35delG (p.Gly12Valfs*2)	Homozygous	20
c.506G > A (p.Cys169Tyr)	Homozygous	3
c.235delC (p.Leu79Cysfs*3)	Heterozygous	1 *
c.299_300delAT (p.His100Argfs*14)	Heterozygous	

* This individual carries both mutations in trans

For the rest of the affected individuals with no *GJB2* mutations, we conducted whole exome sequencing (Fig. 1). Our analysis revealed the presence of 6 homozygous variants located in HL-related genes in 7 patients. Three out of these 6 variants were implicated in previous studies with HL, whereas 3 were not (Table 2). These variants are the 3 new missense variants c.3133 C > T (p.Arg1045Cys), c.934 C > T (p.Arg312Trp), c.6503T > G (p.Leu2168Arg) located in the *TRIOBP*, *CDC14A* and *MYO15A* genes, respectively.

**Table 2:** Homozygous truncating, pathogenic and likely pathogenic variants identified through WES

Case	Phenotype	Gene	cDNA variant	Protein variant	gnomAD frequency	Reported	Polyphen	SIFT
1	Severe to profound	*TMC1*	c.100 C > T	p.Arg34*	0.00001316	Yes	NA	NA
2	Severe to profound	*TMC1*	c.100 C > T	p.Arg34*	0.00001316	Yes	NA	NA
3	Severe to profound	*GPSM2*	c.1055 C > A	p.Ser352*	NA	Yes	NA	NA
4	Severe	*CDH23*	c.6614 C > T	p.Pro2205Leu	0.00001314	Yes	Pr_D	D
5	Moderate to severe	*TRIOBP*	c.3133 C > T	p.Arg1045Cys	0.0004364	No	Po_D	D
6	Moderate to severe	*CDC14A*	c.934 C > T	p.Arg312Trp	0.000008003	No	Pr_D	D
7	NA	*MYO15A*	c.6503T > G	p.Leu2168Arg	NA	No	Pr_D	D

NA: Not available; Po_D: Possibly damaging; Pr_D: Probably damaging; D: Deleterious; NA: Not applicable

Furthermore, we detected seven affected individuals with multiple heterozygous variants within HL-associated genes. Among this group, one patient (Sample # 8, as shown in Table 3) exhibited a potential segregation of two DNA variants in trans within the *OTOF* gene. For the remaining cases, multiple potential pathogenic heterozygous variants were observed across various genes. Moreover, in 12 patients only one heterozygous variant was detected (Table 3) with two nonsense, one frameshift and 9 missense variants. Finally, in 56 patients no potential pathogenic variant was detected in HL-associated genes.

**Table 3:** Heterozygous truncating, pathogenic and likely pathogenic variants identified through WES

Case	Phenotype	Gene	cDNA variant	Protein variant	gnomAD frequency	Reported	Polyphen	SIFT
8	Profound	*OTOF*	c.5159 C > T	p.Thr1720Met	0.0001774	No	Po_D	D
		*OTOF*	c.2374 C > T	p.Arg792Trp	0.0009337	No	Po_D	D
9	Moderate to profound	*USH2A*	c.3812-3_3837dup	p.Met1280*	0.00001314	No	NA	NA
		*PDZD7*	c.166dup	p.Arg56Profs*24	0.00005917	Yes	NA	NA
10	NA	*MYO3A*	c.170 A > C	p.Asp57Ala	0.0009534	No	Pr_D	D
		*PDZD7*	c.543G > A	p.Trp181*	NA	No	NA	NA
11	NA	*USH1C*	c.1477 C > T	p.His493Tyr	0.00005341	No	Pr_D	D
		*OTOG*	c.1214 C > A	p.Thr405Asn	NA	No	Po_D	D
		*DMXL2*	c.796 C > T	p.Arg266Trp	0.00002632	No	Pr_D	D
12	NA	*MYO6*	c.3019 C > T	p.Arg1007Cys	0.00002629	No	Pr_D	D
		*USH1C*	c.121G > A	p.Val41Met	0.00003286	No	Pr_D	D
13	NA	*OTOG*	c.1001 C > T	p.Pro334Leu	0.00006662	No	Pr_D	D
		*MYO7A*	c.247 C > A	p.Arg83Ser	NA	No	Pr_D	D
		*OTOA*	c.3079 C > T	p.Arg1027Trp	0.000076	No	Po_D	D
14	Severe	*MYO6*	c.3019 C > T	p.Arg1007Cys	0.00002629	No	Pr_D	D
		*LOXHD1*	c.1186G > A	p.Glu396Lys	0.00003153	No	Pr_D	D
15	Moderate to severe	*USH2A*	c.3045 C > G	p.His1015Gln	0.00007231	No	Po_D	D
16	NA	*OTOGL*	c.1171 A > T	p.Ile391Phe	0.00003441	No	Pr_D	D
17	Moderate to profound	*MYO15A*	c.5925G > A	p.Trp1975*	0.0005191	Yes	NA	NA
18	Moderate to severe	*USH2A*	c.1367T > A	p.Ile456Asn	0.000007982	No	Po_D	D
19	NA	*TRIOBP*	c.3068 C > T	p.Ala1023Val	0.001046	No	Pr_D	D
20	NA	*S1PR2*	c.985 C > T	p.Arg329Cys	0.00001971	No	Po_D	D
21	Moderate to severe	*CDH23*	c.1691 A > G	p.Lys564Arg	NA	No	Pr_D	D
22	Moderate to severe	*OTOG*	c.4693G > T	p.Gly1565*	NA	No	NA	NA
23	Profound	*MYO7A*	c.1541G > C	p.Ser514Thr	0.00002558	No	Po_D	D
24	Moderate to severe	*USH1C*	c.1597G > A	p.Ala533Thr	0.00007028	No	Po_D	D
25	NA	*TPRN*	c.117del	p.Ala41Argfs*409	NA	No	NA	NA
26	Severe to profound	*OTOGL*	c.3250 A > C	p.Asn1084His	NA	No	Pr_D	D

NA: Not available; Po_D: Possibly damaging; Pr_D: Probably damaging; D: Deleterious; NA: Not applicable

## Discussion

In this study, we investigated 106 sporadic cases affected with HL. To identify the causative mutation, Sanger sequencing of the *GJB2* gene, the most common autosomal recessive non-syndromic hearing loss gene in the UAE population [21] was performed using their genomic DNA. Our analysis revealed that 20 cases were homozygous for the c.35delG mutation, 3 homozygous for the c.506G > A and one patient was compound heterozygous for the c.235delC and c.299_300delAT pathogenic variants. The c.35delG and c.506G > A have been reported previously in the UAE population [21], however this is the first time where we observe the segregation of c.235delC and c.299_300delAT in the UAE. These two mutations are the most common *GJB2* mutations found in the Chinese populations [22] and were detected in neighboring populations [23, 24].

Whole exome sequencing unveiled the presence of the c.100 C > T (p.Arg34*) mutation in two cases, a variant located within the *TMC1* gene. This mutation has been previously documented in numerous affected families hailing from regions such as Algeria, Tunisia, Turkey, Lebanon, Iraq, Iran, Pakistan, and Saudi Arabia [25–30]. Furthermore, it has been identified as a founder mutation in several populations [25, 29, 30].

Another confirmed mutation within our cohort was the nonsense variant c.1055 C > A (p.Ser352*), situated in the *GPSM2* gene. This mutation was previously reported in a Yemeni family with Chudley-McCullough syndrome, characterized by profound congenital sensorineural hearing loss and various brain abnormalities [31].

Additionally, we identified one sample carrying the missense *CDH23* mutation c.6614 C > T (p.Pro2205Leu) in the homozygous state. This variant was also noted in the homozygous state among three consanguineous probands from Qatar and in one sporadic case as a compound heterozygote in the United States [32, 33].

Moreover, our WES analysis unveiled three homozygous variants with the potential to cause disease in three cases. These changes, namely c.3133 C > T (p.Arg1045Cys), c.934 C > T (p.Arg312Trp), and c.6503T > G (p.Leu2168Arg), are located in the *TRIOBP*, *CDC14A*, and *MYO15A* genes, respectively. These genes are associated with three distinct forms of autosomal recessive non-syndromic hearing loss, namely DFNB28, DFNB32, and DFNB3 [34–36].

It is noteworthy that for the *CDC14A* variant (p.Arg312Trp), two mutations involving the amino acid arginine at position 312 have been previously reported: the p.Arg312Gly mutation in an Iranian family with hearing loss and confirmed infertility, and the p.Arg312Gln mutation in a Tunisian family with hearing loss, though fertility was not assessed [37]. In our study, the newly identified mutation, p.Arg312Trp, affects the same amino acid. Interestingly, the patient is a biological father, which indicates that this genetic alteration impacts hearing loss but not fertility. This may suggest that the substitution of arginine 312 with tryptophan retains some enzymatic function. This aligns with prior findings that infertility in some deaf males is linked to specific variants of CDC14A. These variants are associated with the monogenic syndrome Hearing Impairment and Infertile Male Syndrome (HIIMS), caused by inadequate phosphatase activity. In contrast, other variants with residual enzymatic function are implicated in non-syndromic deafness (DFNB32) [37].

Regarding the remaining samples, WES uncovered the presence of more than one heterozygous variant in seven cases. In sample 8, we detected two potential HL-associated variants within the *OTOF* gene in a heterozygous state. It’s noteworthy that compound heterozygous mutations in the *OTOF* gene are frequently observed and have been implicated in many cases of hearing loss [38–44].

Upon evaluating six samples with WES, we discovered multiple potential pathogenic heterozygous variants in genes associated with HL: In sample # 9, we identified two novel truncating duplications in a heterozygous state in two genes: *USH2A* c.3812-3_3837dup (p.Met1280*) and *PDZD7* c.166dup (p.Arg56Profs*24). The proteins encoded by these two genes interact within the ankle region of stereocilia and play a significant role in hair cell development [45, 46]. In previous studies, a similar case was observed for the *PDZD7* gene, where a heterozygous truncating mutation, p.Cys732Leufs*18, occurred alongside another heterozygous frameshift mutation, p.Ala5713Leufs*3, in the Usher protein *ADGRV1* in patients with hearing loss [46]. For samples 10, 11, 12, 13, and 14, we observed multiple potential pathogenic heterozygous variants across HL-associated genes; however, no specific interactions have been reported between the implicated genes. Although numerous potential digenic interactions contributing to HL have been reported [16–18, 46, 48–63], confirming or refuting the role of the multigenic variants observed in patients 9, 10, 11, 12, 13 and 14 requires further segregation analyses and functional tests.

In the case of twelve samples, our WES analysis revealed the presence of only one potential heterozygous pathogenic variant. Among these 12 detected variants, three were truncating DNA changes: the well-known *MYO15A* nonsense variant c.5925G > A (p.Trp1975*), the novel *TPRN* frameshift alteration c.117del (p.Ala41Argfs*409), and the new nonsense *OTOG* variant c.4693G > T (p.Gly1565*). The remaining DNA variations consisted of missense variants, and none of them had been previously reported as causative for HL. It’s worth noting that while most of the genes identified in these 12 patients were associated with autosomal recessive hearing loss, mutations in the *MYO7A* gene have been linked to both autosomal dominant and recessive forms [9].

In our cohort, we found that the *GJB2* gene accounted for 22.6% (24 out of 106) of individuals with hearing loss. This prevalence surpasses the estimates from our previous studies, where it was believed to be 18% [21]. Additionally, our analysis revealed HL-related mutations in the *TMC1*, *GPSM2*, and *CDH23* genes. When combined with data from prior studies, our findings reveal that a total of 29 genes have now been confirmed to play a role in hearing loss in the UAE population [64, 65]. Notably, the most prevalent genes contributing to this condition include *GJB2*, *COL11A2*, *MYO6*, *TMC1*, *TRIOBP*, and *TMPRSS3* [64]. Through WES, we managed to identify confirmed and potential disease-causing genotypes in approximately 25% of the individuals we examined. These findings align with previous research, highlighting the limitations of WES in identifying pathogenic mutations in all affected individuals, particularly in sporadic cases. For instance, a study by Mutai et al. in 2022 successfully identified the responsible gene in 21 out of 72 cases (approximately 29%) using WES [66]. Similarly, Reiss et al. demonstrated that WES analysis of 71 probands with hearing loss revealed pathogenic or likely pathogenic variants in only 21.1% of cases [67].

The high number of cases lacking potential pathogenic variants (~ 55%) may be attributed to the constraints of WES. These constraints include difficulties in achieving complete coverage of coding regions, often due to factors like the high G + C content in sequences, as well as limitations in detecting copy number variants. Additionally, unresolved cases, even sporadic ones, might be due to non-genetic causes of hearing loss, such as childhood infections or acoustic trauma. Other undetectable mutations besides CNVs, such as deep intronic mutations, transposable elements, and epigenetic factors, could also account for these unresolved cases. Determining whether variants are pathogenic, nonpathogenic, or of uncertain significance relies on existing data sources such as ClinVar (http://www.ncbi.nlm.nih.gov/clinvar/) and may evolve as our understanding of the genome advances. Our results are similar to many previous studies where the diagnostic rate of WES was found to be less than 50%. For example the study conducted by Zeng et al. in 2022, which focused on a cohort comprising 152 familial cases with hearing loss, showed a diagnosis rate of merely 18.4%, with 28 out of the 152 cases yielding a definitive diagnosis identified using WES [68]. This result confirms the inherent limitations of WES in achieving a 100% diagnostic rate, particularly in diseases with high genetic heterogeneity like hearing loss.

## Conclusion

In our investigation of 106 sporadic cases of HL, we detected mutations in the *GJB2* gene, notably the prevalent c.35delG mutation. This highlights the significant role of this specific variant and *GJB2* changes in general within the UAE population. Whole exome sequencing unveiled a spectrum of genetic pathogenic and likely pathogenic DNA variations. However, challenges remain, as a substantial portion of cases lack genetic explanations, underscoring the complexity of hearing loss genetics.

## Data Availability

All data generated or analyzed during this study can be obtained from the corresponding author on request.

## Abbreviations

HL: Hearing loss
NGS: Next-generation sequencing
WES: Whole exome sequencing
Indels: Insertions and deletions
CNVs: Copy number variations
UAE: United Arab Emirates
PCR: Polymerase chain reaction
GATK: Genome analysis tool kit
VEP: Variant Effect Predictor
SIFT: Sorting Intolerant From Tolerant
MLPA: Multiplex Ligation-dependent Probe Amplification
